# An Integrin-Targeted, Highly Diffusive Construct for Photodynamic Therapy

**DOI:** 10.1038/s41598-017-13803-4

**Published:** 2017-10-17

**Authors:** Oliver J. Klein, Hushan Yuan, Nicholas H. Nowell, Charalambos Kaittanis, Lee Josephson, Conor L. Evans

**Affiliations:** 1 Wellman Center for Photomedicine, Massachusetts General Hospital, Harvard Medical School, 13th St, CNY149, Charlestown, MA 02129 USA; 2 Department of Radiology, Division of Nuclear Medicine and Molecular Imaging, Massachusetts General Hospital, Harvard Medical School, 13th St, CNY149, Charlestown, MA 02129 USA

## Abstract

Targeted antineoplastic agents show great promise in the treatment of cancer, having the ability to impart cytotoxicity only to specific tumor types. However, these therapies do not experience uniform uptake throughout tumors, leading to sub-lethal cell killing that can impart treatment resistance, and cause problematic off-target effects. Here we demonstrate a photodynamic therapy construct that integrates both a cyclic RGD moiety for integrin-targeting, as well as a 5 kDa PEG chain that passivates the construct and enables its rapid diffusion throughout tumors. PEGylation of the photosensitizer construct was found to prevent photosensitizer aggregation, boost the generation of cytotoxic reactive radical species, and enable the rapid uptake of the construct into cells throughout large (>500 µm diameter) 3D tumor spheroids. Replacing the cyclic RGD with the generic RAD peptide led to the loss of cellular uptake in 3D culture, demonstrating the specificity of the construct. Photodynamic therapy with the construct was successful in inducing cytotoxicity, which could be competitively blocked by a tenfold concentration of free cyclic RGD. This construct is a first-of-its kind theranostic that may serve as a new approach in our growing therapeutic toolbox.

## Introduction

Most current therapeutic regimens for cancer involve the systemic administration of antineoplastic agents that are well known to cause toxic side effects to healthy normal cells and tissue. Efforts to develop molecular targeted therapies have been successful in creating more advanced treatment options that can be tailored to a patient’s specific cancer subtypes^1^. However, many of these new agents still harbor significant morbidities. For example, while antiangiogenic therapies show some promise in slowing the progression of cancer^2,3^, their administration carries significant cardiac risks^4^. An additional degree of selectivity would be highly advantageous in preventing undesired off-target effects.

Photodynamic therapy (PDT) is a light-based treatment modality that offers improved selectivity over traditional chemotherapeutic regimens^5,6^. PDT makes use of molecules known as photosensitizers that generate reactive radical species upon the absorption of specific wavelengths of light^6^. PDT imparts two degrees of selectivity: (1) photosensitizers are preferentially taken up by tumor tissues and (2) the molecules only generate cytotoxic radical species only at the site where light is administered^7^. There are two cytotoxic photochemical mechanisms in PDT: a “Type 1” mechanism where the molecule directly reacts through its excited state to generate reactive radicals species and a “Type 2” mechanism where photosensitizers convert molecular oxygen into highly reactive singlet oxygen. Photosensitizers currently used in the clinic are predominantly “Type 2” molecules. PDT and chemotherapy operate via orthogonal cell death mechanisms; PDT can be successfully used in the treatment of chemoresistant cells^8^ as well as in combination with standard-of-care chemotherapeutics^9,10^. Advantageously, most photosensitizers are also fluorescent, making them potential “theranostics” that can be used for both imaging to identify malignant tissues and therapy to treat the disease.

PDT has been successfully applied to the therapy of numerous malignancies, including head and neck cancer^11^, lung cancer^12^, mesothelioma^13^, and is FDA-approved for the clinical treatment of esophageal cancer^14^. Despite its potential, PDT can suffer from off-target effects. For example, healthy tissues peripheral to tumors can experience undesired photosensitizer uptake, resulting unwanted cytotoxic effects. Additionally, photosensitizers such as Photofrin can localize within the skin leading to sunlight sensitivity following administration. Targeted photosensitizers have shown considerable promise in overcoming theses effects by sparing healthy, uninvolved tissue, reducing systemic or non-tumoral accumulation, and improving selective uptake by targeted tissues A recent example of such an approach was demonstrated by Spring *et al*. using the Type 2 photosensitizer benzoporphyrin derivative (BPD) based targeted agent^15^.

One of the challenges in treating tumors with any front-line chemotherapeutic, radiation, and photodynamic therapy regimens is the microenvironmental factor known as hypoxia^16^. When cells are starved of oxygen, and thus hypoxic, numerous cell survival programs are activated that act in concert to resist conventional therapies, including chemotherapy and radiation therapy. Radiation and current photodynamic therapy regimens are particularly impacted, as they require the presence of oxygen to mediate their cytotoxic effects.

A targeted PDT agent incorporating a Type 1 photosensitizer would therefore be advantageous as it could treat both normoxic and hypoxic tumor regions. Studies have reported that the molecule EtNBS^17,18^, a cationic benzo[a]phenothiazinium molecule, can operate via both photochemical mechanisms and thus impart cytotoxicity even in severely hypoxic environments^19^. EtNBS has been found effective both *in vitro* and *in vivo* ^20^ and diffuses along pH gradients to accumulate in acidic cellular compartments and hypoxic environments^19^. Advantageously, EtNBS has a side-chain that can be readily functionalized^21,22^, enabling its facile conjugation^21^. However, EtNBS is known to have so-called “dark toxicity”, a light-independent toxicity that can manifest when high concentrations of the small cationic photosensitizer are present. It was recently shown that encapsulation of EtNBS within nanoparticles can partially alleviate this toxicity, yet it is unclear if this delivery mechanism will be successful for systemic administration^23^. A mechanism to target EtNBS to cells of interest could avoid this dark toxicity when systemically administered and improve PDT outcome even within hypoxic environments.

One potential avenue for targeting tumor cells is to leverage the integrin overexpression common in many cancers^24^. Integrins are a family of cell surface receptors that are involved in cell-matrix adhesion and contact signaling. These molecules are (type 1) transmembrane heterodimeric glycoprotein receptors composed of alpha and beta subunits, each of which is generated via alternative spicing to create a set of 24 different integrins. High expression levels of key integrins, such as the alphaVbeta3, has been associated increased tumor proliferation in ovarian cancer^24^. A photosensitizer platform that could target cells overexpressing key integrins could prove to be a valuable therapeutic tool.

Here, we present an integrin-targeted EtNBS photodynamic construct created to rapidly diffuse to integrin-expressing targets. EtNBS is conjugated to a central peptide chain that serves as a modular conjugation backbone that hosts two important moieties: 1) a cyclic RGD (cRGD) peptide that provides alpha5/alphaV targeting and 2) a 5 kD polyethylene glycol (PEG) chain that wraps around the construct in aqueous environments via an entropic spring effect^25^ to provide passivation and rapid diffusion^26^. The construct was found to improve the photodynamic radical yield of EtNBS, as well as to facilitate its diffusion through three-dimensional *in vitro* spheroid cultures. The cRGD-PEG-EtNBS construct was found to enter cells in an integrin-dependent manner, and cells could be rescued from PDT-imparted toxicity when competitively inhibited with a tenfold excess of free cRGD. We envision this type of modular construct as a theranostic tool for the imaging and delivery of targeted photodynamic therapeutics in the future.

## Results

### The EtNBS Construct Prevents Photosensitizer Aggregation

**Figure 1:**
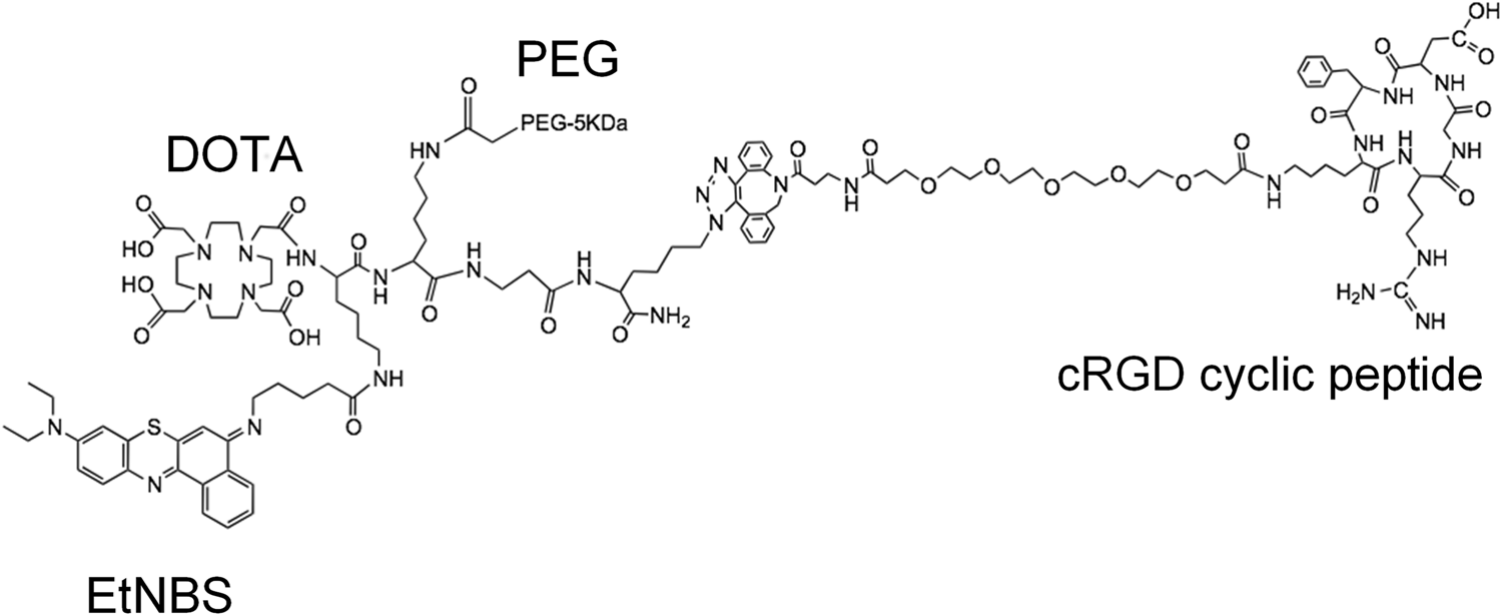
Structure of the EtNBS-cRGD-DOTA-PEG construct. Synthesis of the construct was carried out in two separate synthetic runs to ensure the synthesis could be accurately replicated.

**Figure 2:**
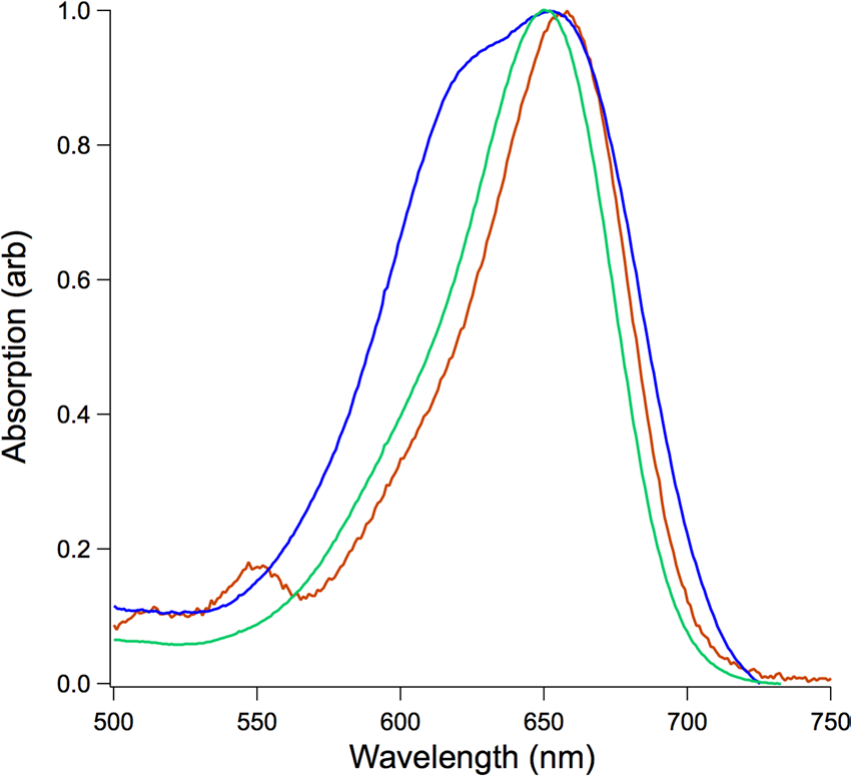
Normalized absorption spectra of EtNBS and the EtNBS-cRGD-DOTA-PEG construct. EtNBS in acidified methanol (green) contains one absorption peak, while the formation of aggregates distorts the spectrum when EtNBS is dissolved in water (blue). The EtNBS construct does not show aggregation in water at a concentration of 87.8 µM (red).

The cRGD integrin-targeted, PEGylated EtNBS construct was synthesized stepwise as described in the methods and characterized by MALDI-TOF mass spectrometry (Fig. 1). Absorption spectroscopy was used to assess the status of the EtNBS moiety to ensure that it was not degraded by the synthetic process. The absorption spectrum of the construct demonstrated all of the features of EtNBS. Interestingly, however, the absorption spectrum of the construct in water different from what is normally observed from free EtNBS (Fig. 2).

The absorption spectrum of EtNBS is typically measured in acidified methanol^19^ to minimize the effects of aggregation. EtNBS, like many benzothiazinium molecules, is known to undergo J-J aggregation in water, which leads to a concentration-dependent blue shift in its spectrum along with ground state quenching^21^. The absorption spectrum of the construct was acquired in water at a concentration of 87.8 µM, where EtNBS would normally be strongly aggregated, did not have this aggregation-induced blue shift, and instead appeared similar to that of unaggregated EtNBS in acidified methanol (Fig. 2). This anti-aggregation effect can be understood given the structure of the construct - EtNBS is bound within the core of the construct and surrounded by a long 5 kDa PEG that acts to reduce aggregation. To determine if this anti-aggregation effect could arise solely due to PEGylation, a sidechain-carboxylated derivative of EtNBS (EtNBS-COOH) was reacted with a heterobifunctional 5 kDa amino-methoxy PEG to create a simple EtNBS-PEG conjugate. The absorption spectrum of this EtNBS-PEG conjugate was observed to be identical to that of the construct (Supplementary Fig. 3), indicating that the long PEG chain is indeed the structural component that acts to isolate individual EtNBS molecules from each other and prevent aggregation.

### The EtNBS Construct Enables Greater Photosensitizer Reactive Oxygen Species Yield

Given that the construct reduces aggregation, it would stand to reason that the photodynamic yield of the construct might be enhanced relative to free EtNBS in water. To test this hypothesis, two standard fluorogenic assays were employed to report the generation of reactive oxygen species (ROS): singlet oxygen sensor green (SOSG) and hydroxyphenyl fluorescein (HPF). It should be noted that while the primary ROS detected by these two indicators are singlet oxygen and hydroxyl radicals, respectively, the sensors are not entirely selective to these two species^27^. These two indicators thus provide here a relative comparison of ROS generation between the EtNBS construct and free EtNBS.

**Figure 3:**
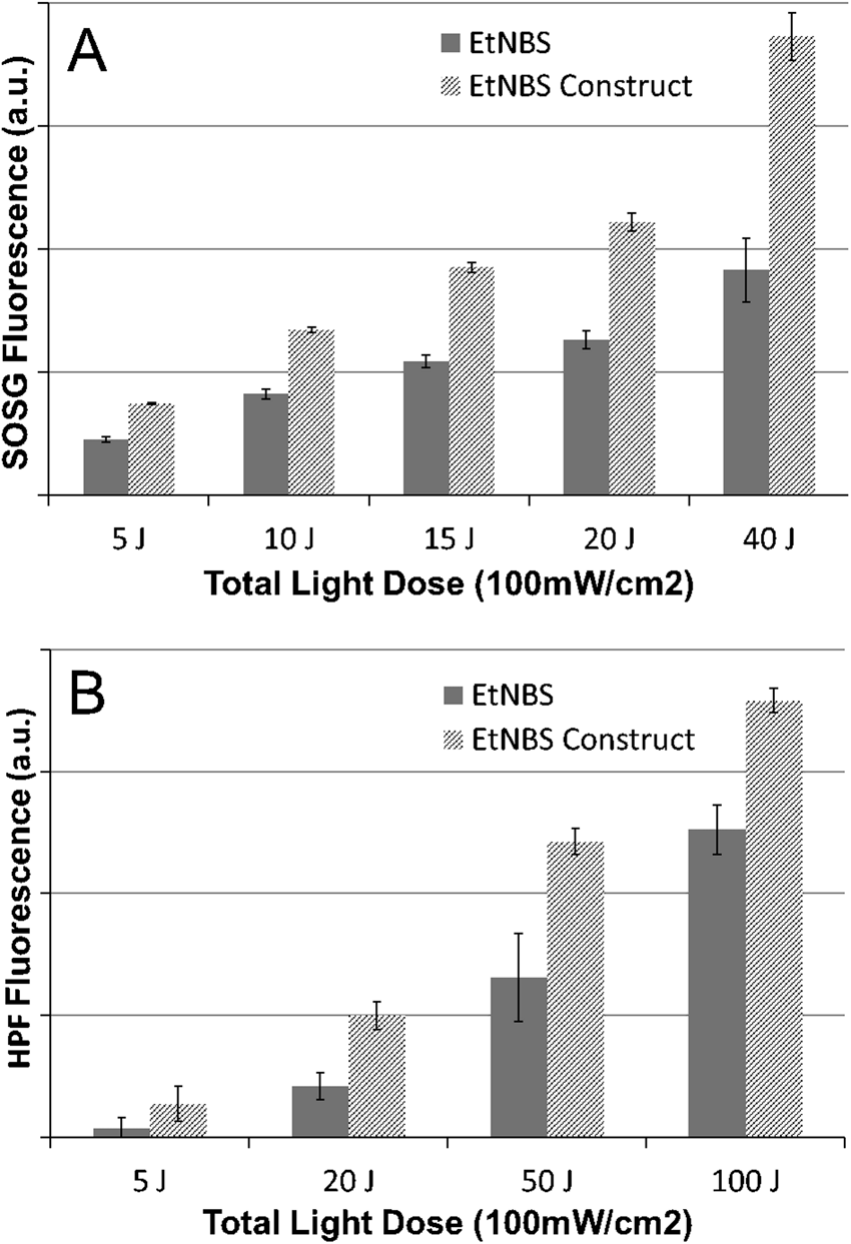
The EtNBS construct create greater levels of reactive oxygen species than free EtNBS under the 100 mW/cm^2^ irradiance conditions. The EtNBS construct is found to generate greater singlet oxygen sensor green (SOSG) signals at all tested light doses (**A**). Similarly, the reactive oxygen levels indicated by HPF were all greater for the EtNBS construct at all light doses (**B**). All experiments were carried out in triplicate; the data presented is the mean of the measurements with the error bars are derived from the standard deviation.

When used at equimolar concentrations in water, the EtNBS construct was found to have considerably greater photodynamic yield than EtNBS as observed by both SOSG and HPF (Fig. 3). ROS generation by this construct as measured by SOSG was greater than that of free EtNBS at all light doses tested (5–40 J/cm^2^), with the yield of singlet oxygen being nearly 100% greater at the highest light dose. Similarly, measurements with HPF over a larger light dose range (5–100 J/cm^2^) showed considerably greater photodynamic activity across all light doses, with a two-fold enhancement of HPF fluorescence with a dose of 50 J/cm^2^. It is well known that EtNBS molecules can self-quench via ground state quenching when in close proximity. In fact, sensors and theranostics have been developed based on this phenomenon^21^. The isolation of each EtNBS molecule that is provided by the construct prevents aggregation-induced intramolecular quenching, which enables greater ROS yields.

### Cellular Uptake of the EtNBS Construct is Dependent on alpha5 Integrin

Free EtNBS is known to enter and become retained within cells via an ion trapping mechanism that occurs within the low-pH endosomal and lysosomal environments once the molecule passively diffuses across cellular membranes. This localization is considered advantageous for PDT, as lysosomal photosensitizers such as EtNBS have been found capable of inducing apoptosis while simultaneously preventing autophagic rescue^28^. Based on prior studies, the integrin-targeted construct was expected to enter cells via an alpha integrin receptor-mediated endocytic mechanism^26^ whereby it would ultimately reside within endosomal and lysosomal compartments. Cellular uptake experiments making use of LysoTracker Green confirmed that the EtNBS construct could be found exclusively within lysosomes 1.5 hours post-administration (Supplementary Fig. 4).

**Figure 4:**
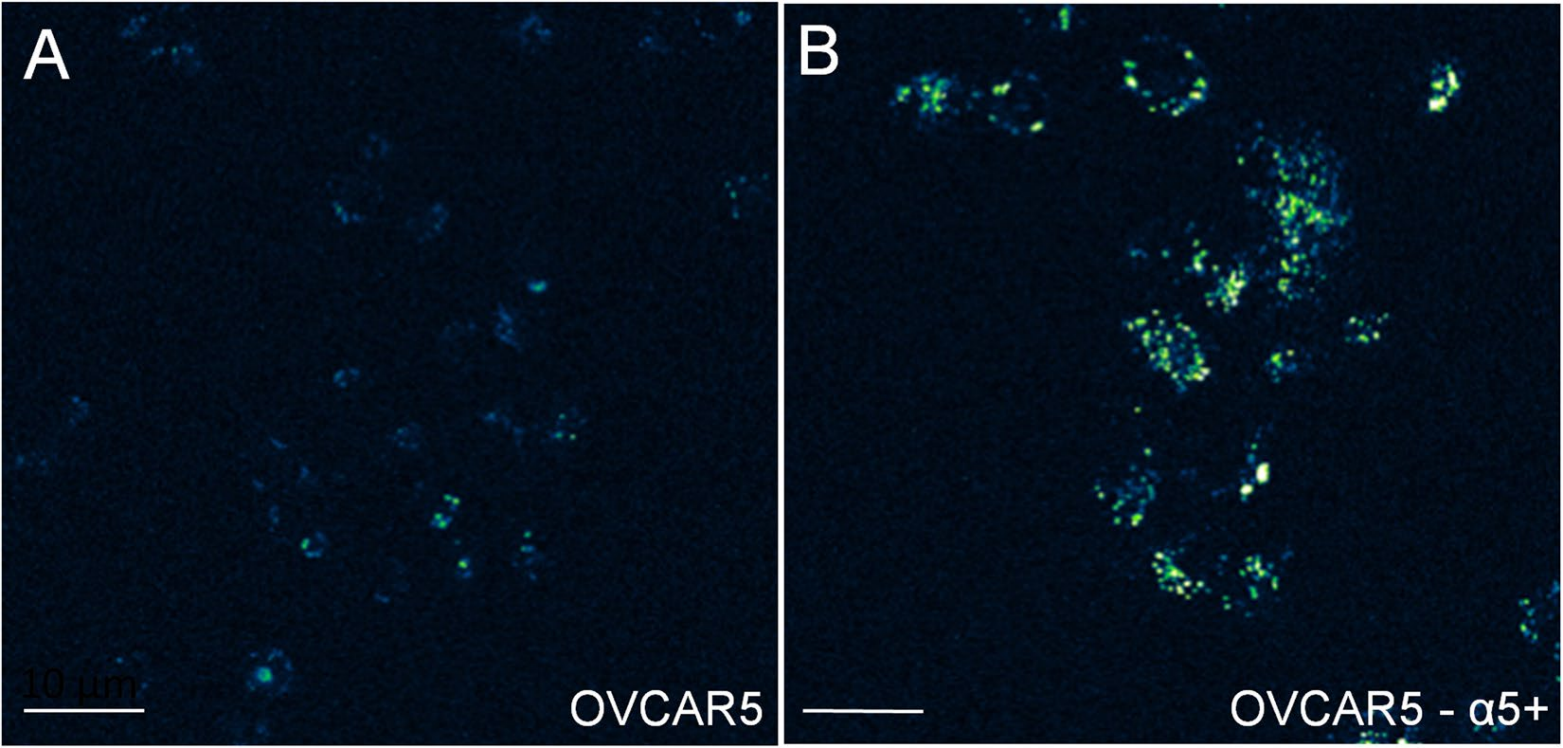
The EtNBS construct experiences greater uptake in cells that overexpress the α5 integrin. To better show the range of construct uptake, the images are false colored in a blue-green-white color scale. The contrast in both images arises from the native near-infrared fluorescence of the EtNBS molecule when excited at 635 nm. Both the normal OVCAR5 (**A**) and an OVCAR5 cell line engineered to ectopically overexpress the α5 integrin (**B**) were incubated with the EtNBS construct. The cell line with greater integrin expression demonstrated greater uptake of the construct via confocal fluorescence microscopy (**B**).

To determine if the uptake of the construct is dependent on the cell surface expression levels of integrin, specifically the alpha5 integrin targeted by the RGD sequence, both OVCAR5 and a genetically modified OVCAR5 cell line were incubated with the construct. The latter genetically-modified OVCAR5 line was specifically developed to ectopically overexpress the alpha5 integrin, which was previously shown to accelerate the invasion of cellular spheroids into model peritoneal surfaces^29^. Confocal fluorescence microscopy was used to quantitatively visualize the EtNBS construct via its 670 nm fluorescence emission peak. As can be seen in Fig. 4, the uptake of the construct is significantly greater in the alpha5 integrin ectopically overexpressed cell line. While the degree of uptake is increased in the overexpressing cells, the cellular localization pattern is observed to be the same. It is worth noting that while the overexpressing cells do take up more of the targeted PEGylated construct than the unPEGylated one, in both cases the photosensitizer concentration due to construct uptake within the cells is many times lower than that of administered free EtNBS due to different internalization mechanisms (receptor-mediated versus passive diffusion that lacks specificity). In the case of receptor-mediated trafficking, the cellular concentration of monovalent ligands, like the construct, is dictated by the internalization rate and abundance of its corresponding receptor. As the concentration of the construct is typically far greater than that of the receptor abundance, the binding of the agent to receptor can be considered essentially limited to pseudo first order with the receptor abundance. This is important as the uptake of photosensitizers into cells have been found to correlate strongly with readouts of PDT efficacy, such as ROS production and photosensitizer photobleaching, and thus the ultimate photocytoxicity of the agent^6^.

**Figure 5:**
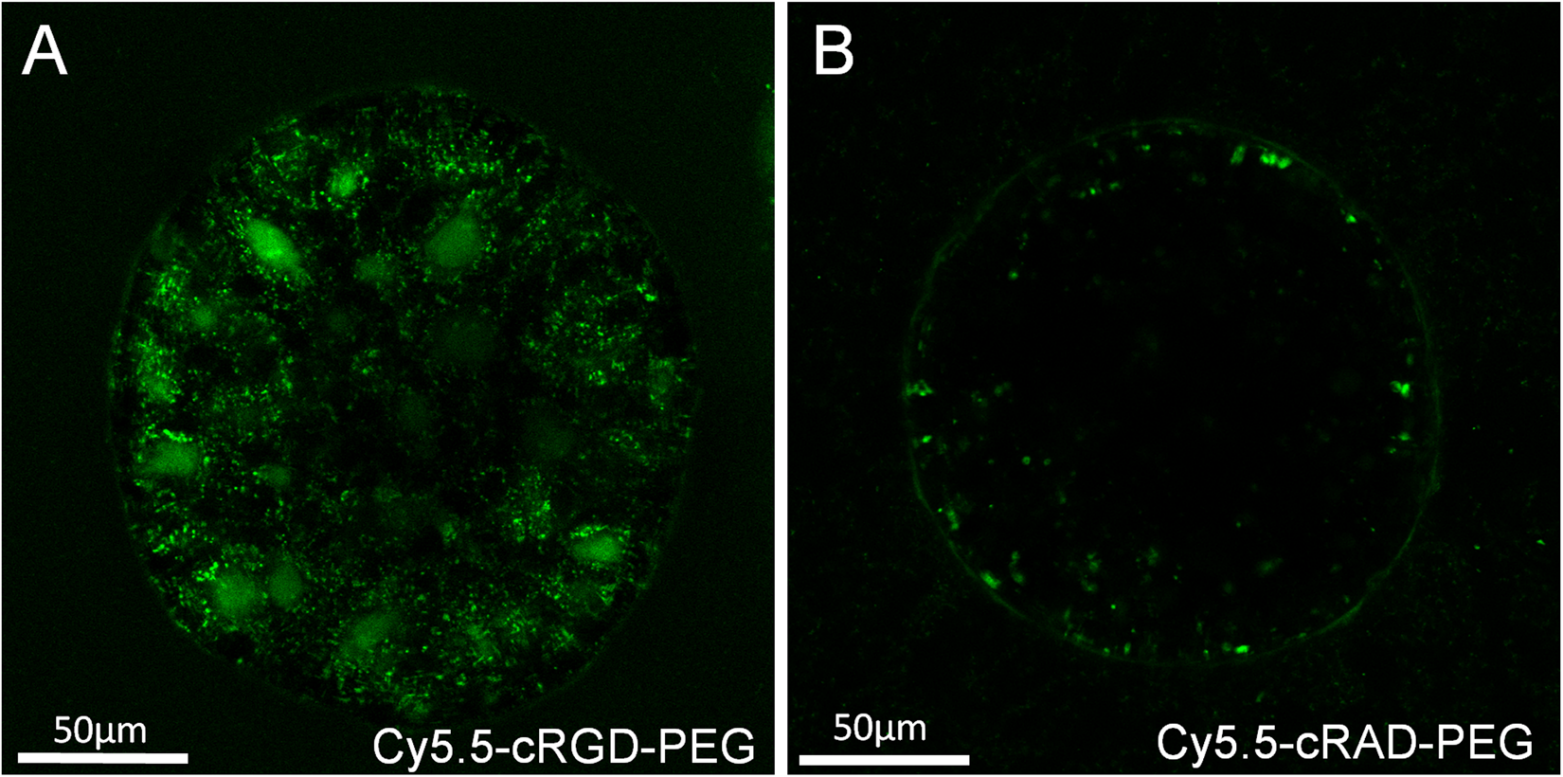
The construct is taken up into cells specifically via its integrin targeting. While the construct incorporating cRGD sees widespread uptake in 3D ovarian cancer spheroids (**A**), the same construct containing the cRAD moiety experiences only weak nonspecific uptake (**B**).

The RGD sequence is highly specific for integrins, such that a change in only one amino acid eliminates binding specificity^26,30^. To test if the uptake of the construct is indeed dependent on the cRGD moiety, an off-target cyclic peptide was incorporated into the same construct: cyclic RAD (cRAD). So as to probe the specificity of the cRGD and cRAD constructs in the parent OVCAR5 line, a 3D *in vitro* spheroid model was employed that replicates many of the features found *in vivo*, in particular the presence of extracellular matrix^9,19,31,32^. As these spheroid cultures can grow to sizes in excess of 750 µm in diameter, alternative cRGD and cRAD constructs were synthesized that instead employed Cy5.5 dyes, whose greater fluorescence quantum yield enabled improved imaging over those based on the weaker EtNBS fluorescence emission. The difference between these two Cy5.5-cRGD and Cy5.5-cRAD constructs can be seen in Fig. 5. While the cRAD-based construct experiences minimal uptake within the cells of the spheroid, the on-target cRGD construct is able to enter each cell and localize to lysosomes. This result underlines the importance of targeted agents: even the PEGlyated, off-target cRAD construct experiences a degree of nonspecific binding to the extracellular matrix surrounding the spheroid. It should be noted that when administered to monolayer cell cultures, only the Cy5.5-cRGD showed any level of uptake into cells.

### PEGylation improves cellular uptake and reduces nonspecific binding

**Figure 6:**
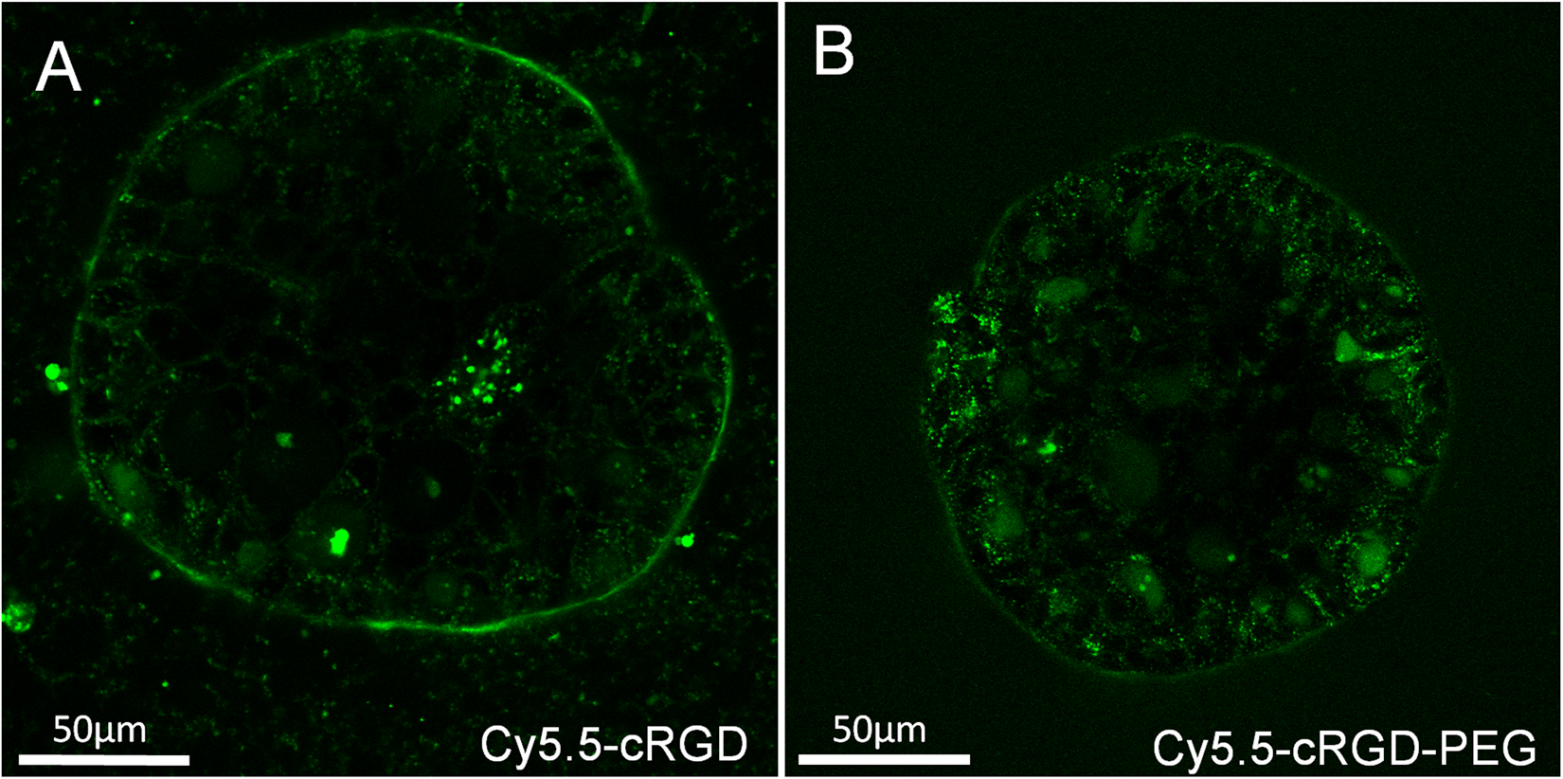
PEGylation dramatically improves the penetration of the construct into 3D tumor spheroids. The construct lacking the PEG chain only reaches cells at the nodule periphery and is mostly bound to the layer of extracellular matrix that surrounds the spheroid (**A**). The PEGylated construct, on the other hand, has minimal extracellular matrix binding and is taken up within cells throughout the spheroid (**B**).

Previous studies exploring PEGylated constructs noted that the addition of large molecular weight linear PEGs enabled the molecules to rapidly diffuse *in vivo* at a rate far exceeding that of their non-PEGylated analogs^26^. To explore this difference in the OVCAR5 3D model culture, both PEGylated and non-PEGylated Cy5.5-cRGD constructs were synthesized. As can be seen in Fig. 6, the PEGylated construct’s passivation and reduced nonspecific binding to extracellular matrix is clearly advantageous, with the construct being taken up by cells throughout the spheroid. The non-PEGylated construct, in contrast, can be seen only in the cells at the surface of the spheroid and is bound to a thin ring of extracellular matrix secreted by the spheroid^19^. The observed uptake of the large PEGylated photosensitizer construct through all the cells of the spheroid is remarkable, in particular in comparison to the known problems in solid tumor delivery of many photosensitizers^33^. Most photosensitizers cannot penetrate significantly through cellular layers and instead localize close to the vasculature. While this perivascular localization can be advantageous for triggering “vascular shutdown” post-treatment, thereby starving tumors of oxygen and nutrients, vascular destruction has recently been shown to have unintended consequences, such as upregulation of hypoxia and other survival program that enable some tumor cells to survive^34^. A photosensitizer that diffuses throughout all cells within a tumor could prove capable of killing a far greater tumor fraction than currently available PDT agents.

### The Targeted EtNBS construct is an effective photodynamic therapy agent

**Figure 7:**
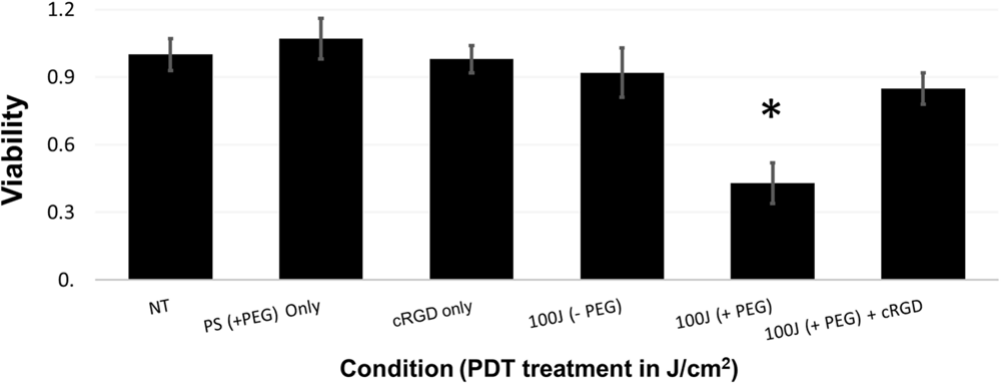
The cRGD-EtNBS-PEG construct is an effective PDT agent. No treatment cells (NT), cells given the cRGD-EtNBS-PEG construct only (PS only), and cells given cRGD only were not exposed to light. Cells treated with unPEGylated cRGD-EtNBS (−PEG) or the full construct EtNBS-cRGD-PEG (+PEG) were irradiated with 660 nm light at 100 mW/cm^2^ for a total dose of 100 J/cm^2^. The far-right bar corresponds to cells first pre-incubated with free cRGD before treatment with EtNBS-cRGD-PEG and subsequent irradiation (100 J (+PEG) + cRGD). Percentage cell viability as determined by the MTT assay is plotted for each group. All experiments were carried out in triplicate; the data presented is the mean of the measurements with the error bars are derived from the standard deviation. The 100 J +PEG treatment condition was found to be statistically significant against all other conditions by t-test (p < 0.05).

To assess the ability of the construct to carry out PDT, monolayer OVCAR5 cells were incubated with the EtNBS construct and illuminated with 660 nm light (Fig. 7). Cellular viability was read using the MTT assay 24 hours post-treatment, which has been shown to be a reliable means of measuring cellular death following photodynamic therapy^35,36^. No “dark toxicity”, defined as toxicity in the absence of treatment light, was found at the 5 µM administration concentration of the EtNBS construct when compared to no-treatment wells. This protective effect stands in stark contrast to free EtNBS, which has been observed to cause a 70% drop in viability at a 5 µM concentration due solely to its dark toxicity.

Prior to carrying out this experiment, small scale experiments were used to determine if the constructs (−PEG, +PEG) gave any measurable toxicity without light. These compounds were found to be equally harmless, and therefore the -PEG compound was not included in the final large-scale experiment. Furthermore, for the combination of +PEG+cRGD, the decision was made to not include a light-only this control for the following reason: cRGD when administered solely to cells did not result in any measured toxicity. In the +PEG+cRGD experiment, cRGD is added sequentially prior to the +PEG construct at a tenfold molar excess; the sequential addition and molar excess results in very little of the +PEG binding to cells. As the +PEG construct also causes no measurable toxicity, and as little or no +PEG construct will likely bind in the presence of cRGD, this control was not included.

Cells treated with the full EtNBS-cRGD-PEG construct demonstrated a 60% reduction in viability with a 100 J/cm^2^ light dose. Observation of the treated cells with phase contrast microscopy revealed cellular morphology consistent with apoptosis. In contrast, cells treated with the non-PEGylated, EtNBS-cRGD construct showed a complete lack of cell death. This result strongly correlates with the results shown in Fig. 5, where it was shown that PEGylation was necessary to achieve cellular uptake. Importantly, a set of wells was pre-treated with just the cRGD moiety used in the construct alone at a concentration of 50 µM (tenfold molar excess). These cells were then washed and treated with the cRGD-PEG-EtNBS construct. As can been seen from Fig. 7, preincubation with cRGD protected the cells against PDT, likely by blocking the binding by competitive inhibition of the construct with integrins and thus preventing receptor binding and cellular uptake. This protection against PDT provides clear evidence of integrin target specificity.

It is worth noting that the 100 J/cm2 light dose is higher than what is typically used for EtNBS PDT; for example, a recent paper using EtNBS achieved a similar level of cell killing using a 10-fold lower EtNBS dose (500 nM) at a 20-fold lower light dose (5 J/cm^2^)^19^. This is true even though the construct is more efficient in the generation of reactive radical species. This difference in light dose between the free molecule and its targeted construct, however, is not entirely surprising; similar higher light doses have been found necessary in immunoconjugates based on the benzoporphyrin derivative photosensitizer^15^. It is hypothesized that the higher photosensitizer and light doses are required for two main reasons. Firstly, free EtNBS easily diffuses across cellular membranes and becomes trapped in lysosomes, while the construct is taken up only through a receptor-mediated process. The difference in photosensitizer uptake is large, with free EtNBS experiencing up to two orders of magnitude greater uptake over the construct. Even cells expressing high levels of surface alpha5 integrin will not be able to import concentrations of EtNBS comparable that of administered free EtNBS. A second reason for the higher required photosensitizer and light doses might be related to the localization of EtNBS within the lysosome. We postulate that when free EtNBS is administered, it accumulates within the lipid membrane of the lysosome, where PDT causes the generation of ROS and rupture of the organelle. The localization of the EtNBS construct within the lysosome is not entirely known, but the construct may still be bound to its integrin receptor. As ROS have short diffusion lengths due to their high reactivity, one might hypothesize that ROS created by the construct react primarily within their local protein-bound environment, thereby causing less damage to the host cell at large.

## Discussion

The integrin-targeted, PEGylated EtNBS construct was found to be an effective photosensitizer for photodynamic therapy *in vitro*. The molecule’s uptake and ultimate PDT efficacy were observed to be dependent on the level of available cell surface alpha5 integrin receptors. PEGylation of the construct considerably improved its uptake into individual cells as well as into *in vitro* 3D tumor spheroid models. The ROS yield of EtNBS within the construct was additionally found to be enhanced by PEGylation, which prevents photosensitizer aggregation in aqueous biological solutions/environments. When used *in vitro*, the construct was able to achieve a 60% level of cell death at a light dose of 100 J/cm^2^.

In this work, we focused on how subtle modifications on a compound can improve its therapeutic activity and specificity. The construct described provides an attractive alterative to regulatory approval, as opposed to other strategies. For instance, although the scientific community focused on the development of nanoparticle-based photosensitizers, the large-scale synthesis, characterization and approval by the FDA can be challenging, due to the inherent complexity and composition of the nanoparticle. From a mechanistic standpoint, these approaches require degradation of the nanoparticle within the endosomal compartment, release of the photosensitizer with the endosomal/lysosomal vesicle, and finally escape to the lysosome. In contrast, our approach relies on clinically approved building blocks, with the construct readily and rapidly diffusing from endosomal compartments to the cytoplasm, in order to exert its cytotoxic activity. Furthermore, for poorly vascularized tumors, our macromolecular construct can provide improved uptake and renal clearance, as opposed to nanoparticle agents, which require extensive vascularization, and tend to be cleared through the hepatobiliary route^37–39^. When compared to other macromolecular constructs, such as antibody-drug conjugates^40^, our approach provides a more robust system, which yields more affordable targetable drugs, with improved solubility, facile characterization and modularity for theranostic applications.

The photodynamic efficacy of the construct could be improved via several different methods. The inclusion of a single EtNBS molecule could be expanded to a chain of many EtNBS moieties. For example, a linear lysine peptide chain could be used to conjugate multiple EtNBS-COOH molecules. Given the propensity of EtNBS to undergo ground-state quenching, a high-persistence length peptide sequence could be constructed that would space out individual EtNBS molecules for greater efficacy. Another method worth exploring would incorporate a cleavable construct that would release EtNBS once the construct reaches the low-pH lysosomal environment^41^. The use of other photosensitizers can and should also be explored. It is worth noting that while not all fluorophores are robust enough to survive synthesis of the construct^26^; EtNBS is particularly stable in the acidic synthetic conditions. Nonetheless, the use of other photosensitizers can and should be explored.

It is interesting to note that the PEG, despite being of large molecular mass compared to EtNBS, did not seem to impart a negative effect on the reactive oxygen yield of the construct. This can potentially be understood by the nature of PEG in water: the molecule is known to form an entropic spring in water^25^ such that it will wrap around the core of the construct. The PEG takes up considerably greater volume that its mass would suggest via creating pockets of solvated water^26^. It is thought that this large expanded structure of solvated water would not act to impede the diffusion of ROS, which can readily pass through dynamic PEG structure. This phenomenon has been known in the oxygen-sensing community, where PEGs have been used to passivate porphyrin oxygen sensors without any noticeable impact on molecular oxygen diffusion^42^.

Given the integrin-specificity of the targeted EtNBS construct, we envision several potential use cases. In preclinical research settings, the EtNBS construct and similar targeted derivatives could be highly useful as a theranostic tool for visualizing and treating specific cells within the cancer milieu. The DOTA moiety included in the construct could be useful for PET or SPECT based biodistribution measurements, not only in preclinical settings but also in clinical applications as a theranostic^26^. The modular nature of the construct lends it to the use of a variety of peptide sequences, including aptamers.

For clinical application, these constructs could be of great use given their ability to rapidly diffuse through tissue. A photosensitizer capable of diffusing throughout a tumor could potentially improve therapeutic efficacy, especially constructs that make use of Type 1 photosensitizers to overcome the limitations imposed by hypoxic microenvironments. This is particularly true for red-sensitive photosensitizers like EtNBS, which can be one-photon excited deep from the tissue surface^6^. Due to reduced blood and tissue absorption, as well as reduced scattering in the turbid tissue environment, red light can readily penetrate at high intensity 2–3 millimeters from the surface of tissue^43^ enabling photocytotoxicity throughout large tumor masses. This is highly advantageous for reaching hypoxic environments within tumors that may lie far from the parenchyma. For tumors that lie within the body, the photodynamic therapy with red light is still advantageous and can be delivered either via endoscopes or via fiber optic tools. For example, in a recent BPD-PDT study treating pancreatic cancer, a diffusing fiber placed via a needle was capable of delivering light throughout large tumors to create a wide zone of necrosis^44^. The ability of the EtNBS construct to diffuse throughout tumors, bind to specific cellular targets, and be photoexcited by red light over large volumes could be advantageous for the treatment of normally resistant cellular subtypes that are not normally accessible to most therapeutics.

Applications could include these EtNBS constructs as part of a combination regimen, in particular for targeting cells within specific microenvironments or certain types of persistent cells that are found to survive standard therapeutic regimens. This type of combination approach would also play to a central strength of PDT, in that the therapy overcomes many cellular resistance mechanisms by directly triggering apoptosis and necrosis. While not used in this study, the DOTA moiety could also be used to chelate radiotherapy agents. Photodynamic therapy is known to potentiate and synergize with radiotherapy; a construct with both PDT and radiotherapy modalities could be of use for more effective targeted cell killing.

## Methods

### Synthesis of the EtNBS Construct DOTA-Lys(ETNBS)-Lys(PEG5K)-β-Ala-Lys(DBCO-PEG4-cRGD)

The synthesis of multifunctional targeting PDT agent, DOTA-Lys(ETNBS)-Lys(PEG5K)-β-Ala-Cys(DBCO-PEG4-cRGD), was carried out by following a previously reported procedure^26^ with the modification of ETNBS as the new fluorochrome. In brief, the procedure includes: (1) manually building up of peptide scaffold with solid phase synthesis through a Fmoc-protected amino acid strategy activated by PyBOP in DMF in the presence of DIPEA; Fmoc deprotected by piperidine; and peptide cleavage by TFA with small amount of water, triisopropylsilane, and ethylenedithiol; (2) attachment of cRGD as the vehicle for molecular targeting through a copperless click chemistry at ambient temperature; (3) grafting a single chain 5 kDa PEG onto the free amine on the peptide scaffold for regulating the pharmacokinetics of the whole agent. All of the compounds were purified by HPLC and characterized by mass spectroscopy. The purity of the final product was analyzed by FPLC. (Detailed protocol, see Supplemental Materials & Methods).

### Cell Culture

All OVCAR5 cells were cultured as described previously^22^. The OVCAR5 parent (Fox Chase Cancer Institute) and alpha5 integrin overexpressing (Joan Brugge, Harvard Medical School) lines were maintained in complete RPMI 1640 media (Corning CellGro) supplemented with 10% heat-inactivated fetal bovine serum (FBS, Life Technologies) and 1% penicillin-streptomycin (5000 IU/mL, CellGrow, MediaTech). Cells were passaged every 3–4 days with 0.05% trypsin (CellGrow, MediaTech). To aid in the detachment of the cells from the plasticware, trypsin was added after a 20 minute incubation with calcium- and magnesium-free Dulbecco’s phosphate-buffered saline (DPBS, CellGrow, MediaTech). For imaging experiments, cells were plated on coverglass-bottomed dishes (*In Vitro* Scientific).

### 3D culture

Three dimensional OVCAR5 cultures were grown as previously described^31^. Briefly, OVCAR5 cells were seeded onto a bed of growth factor reduced (GFR) Matrigel (BD Biosciences) and allowed to grow for a period of 13 days to reach spheroids with an average diameter of approximately 200 µm. 3D cultures were administered 1 µM solutions of the different Cy5.5-peptide conjugates in complete culture medium, after a DPBS washing step. After a 5 hour incubation in the 37 °C incubator under 5% CO_2_, the treatment medium was removed and replaced with DPBS. After repeating this step for a total of two DPBS washes to remove residual dye not taken up by nodules, 3D cultures were returned to the matrigel-supplemented growth media in which they are customarily maintained for imaging sessions.

### Microscopy

All imaging experiments were carried out on an Olympus FV1000 confocal microscope with a stage-top incubator (Prior) to maintain humidity, 37 °C temperature, and 5% CO_2_ during imaging sessions. For each sample, imaging was restricted to 15 minutes from the time cells were removed from the incubator to avoid any artifacts related to cell death. Image acquisition parameters were chosen which captured signal from both samples/cell lines without any saturation of signal and were kept uniform between samples to allow for direct one-to-one comparison of signal. These parameters were also used to image cells treated with known concentrations of EtNBS, to allow for relative comparison of uptake against known references. For every imaging experiment and conditions, a minimum of three areas of interests were collected and analyzed.

### Image Processing

All images were processed using ImageJ (NIH) and the LOCI Bioformats Java package. To accentuate differences in measured intensities, images were false colored. No other processing or manipulations of the images (e.g. brightness/contrast, thresholding, masks) were utilized.

### Spectroscopy

Spectra were acquired using a home-built spectrometer that made use of a white light source for absorption measurements and a 655 nm LED for EtNBS excitation. Transmitted or absorbed light was collected and fiber-delivered to a QE65000 spectrometer (Ocean Optics) for spectral acquisition.

### Monolayer Viability Studies

The MTT assay was used to evaluate cellular viability of PDT-treated monolayer cultures. The MTT assay operates via the conversion of the Thiazolyl Blue Tetrazolium Bromide reagent into purple-colored Formazan via mitochondrial esterases, and thus serves as a metric of cellular viability via mitochondrial activity. The MTT reagent (Thiazolyl Blue Tetrazolium Bromide, Sigma) was administered to cells in warmed complete cell culture media at a concentration of 1 mg/mL. Growth medium was gently aspirated from wells so as not to remove weakly adhered cells and was replaced with MTT media solution (160 µL). Cells were then returned cells to the 37 °C incubator and kept under 5% CO2 for 60 minutes. After incubating cells, the MTT treatment solution was carefully and completely aspirated from wells, with DMSO then added (180 µL) to extract cellular Formazan. After incubating samples for 15 minutes at room temperature while periodically rocking plates to completely and uniformly dissolve Formazan crystals, Formazan-containing DMSO was aliquoted (50 µL volumes from each well in triplicate) into a 96-well plate alongside wells containing an equal volume of DMSO to be used for background subtraction. The absorbance of Formazan at 540 nm was then measured using a SpectraMax M5 (Molecular Devices) plate reader.

After averaging measured absorbance values, background-subtraction was carried out and the standard deviation of the experimental values was calculated. For each individual sample group or treatment condition, the background-subtracted average absorbance value was normalized to that of the untreated (“NT”) control group to obtain the normalized cellular viabilities. The MTT viability assay was used to evaluate cellular viability of monolayer cultures 24 hours after carrying out PDT, as previous studies in our lab have shown that cellular processes resulting from therapy are complete within that time. All measurements were performed in triplicate to ensure high quality data collection.

### Assaying Reactive Oxygen Species Generation

HPF (H36004) and SOSG (S36002) were purchased from Life Technologies and were used as directed by the manufacturer’s protocol. Working solutions were prepared at 10 µM in DPBS or 5 µM TRIS buffer (pH 7.5) for HPF and SOSG, respectively, using 10 µM concentrations of the appropriate photosensitizer. All sample irradiation was carried out using 660 nm-centered LED-generated light (M660L3, Thorlabs) at an irradiance of 100 mW/cm^2^. Irradiation of 80 µL sample volumes was carried out in clear plastic 96-well plates, with the emission of the ROS probes measured with a plate reader (SpectraMax m5, Molecular Devices). Experiments were performed in triplicate and experimental values (N = 3) were averaged and background-subtraction was performed.

### Imaging Integrin-Dependent Uptake of EtNBS-cRGD-DOTA-PEG in OVCAR5 Monolayer Cultures

Stably transfected OVCAR5 cells overexpressing the alpha5 integrin as well as wild-type OVCAR5 cells were plated as described above in glass-bottom 35 mm culture dishes (P35G-0.170-14-C, MatTek) at a density of 200,000 cells per dish 24 hours prior to the start of experiments. Cells were administered the photosensitizer at a concentration of 10 µM in warmed complete cell culture medium and incubated for 3 hours in a 37 °C incubator under 5% CO_2_. Samples administered EtNBS at known concentrations (250 nM and 500 nM) in complete culture medium were used as references for comparison. Photosensitizer-containing media solution was aspirated and replaced with fresh culture medium for imaging. Cells were returned to the incubator for a brief period to recover before the imaging session (15–45 minutes).

### Photodynamic Therapy

Monolayer cultures were plated in clear plastic 48-well plates as previously described 24 hours prior to experiments^22^, at a density of 40,000 cells per well. All steps involving the manipulation of photosensitizers were carried out under subdued lighting to the extent that it was possible to avoid artifacts from unintentional photodynamic effects. Wells within each dish were spaced far apart from each other to ensure minimal light administration cross-talk. All manipulations of media or other culture liquids were done manually, specifically avoiding the use of vacuum aspiration techniques to avoid the unintended removal of weakly adhered cells before, during or after therapy.

Sequential blocking of integrins with cRGD peptide was achieved by administering a 10-fold excess of cRGD peptide (50 µM) in warmed DPBS to cells and incubating for a duration of 3 hours at 37 °C in a 5% CO_2_ incubator. Blocking solution was removed and replaced with photosensitizer-containing treatment media without performing a washing step and cells were immediately returned to the incubator for the appropriate time.

Concentrated stock solutions of photosensitizers were diluted in and administered to cells in warmed complete cell culture media. Stock solutions of photosensitizer in DMSO were diluted such that the final DMSO concentration was less than 1% by volume of the treatment solution. Integrin-dependent PDT efficacy was evaluated using 10 µM photosensitizer doses. The comparison of PEG-dependent enhancement in PDT was evaluated using 5 µM photosensitizer doses. For the duration of the 3 hour photosensitizer incubations, cells were kept under 5% CO_2_ in a sterile 37 °C incubator unless otherwise stated. Following the incubation period, photosensitizer-containing treatment media was aspirated from wells and replaced with fresh complete media (warmed) and subsequently irradiated using a custom-built PDT illumination setup. For large-scale PDT experiments and/or experiments with the administration of each light dose was long, the administration of photosensitizers was staggered to ensure uniformity in the drug-light interval across all samples.

Irradiations were carried out at RT under ambient room oxygenation, the duration of which did not exceed 1 hour, which we have previously shown has no significant/detectable effect on cellular viability or treatment response^22^ Non-irradiated control groups were also kept out of the incubator for the duration of the illuminations to maintain uniform environmental conditions across all sample groups throughout the entire experiment. For the studies reported here, individual sample wells were independently irradiated with 100 mW/cm^2^ light generated by the 660 nm-centered LED light source described above. The duration of light exposure, and thus total light dose, as was controlled using a programmable shutter. Following irradiation, samples were returned to the 37 °C incubator and kept under 5% CO_2_ for 24 hours before the evaluation of cellular viability with the MTT assay. All conditions were carried out in triplicate.

### Data Availability

The datasets generated and analyzed during the current study are available from the corresponding author on reasonable request.

## Electronic supplementary material

Supplementary Information
