# Supplementary material for: An Integrin-Targeted, Highly Diffusive Construct for Photodynamic Therapy
Source: Sci Rep. 2017 Oct 17;7:13375. doi: 10.1038/s41598-017-13803-4 (PMC5645319; doi:10.1038/s41598-017-13803-4)
Supplement: Supplementary file 1 — Supplementary Information [file 41598_2017_13803_MOESM1_ESM.pdf]

Supplementary Information

An Integrin-Targeted, Highly Diffusive Construct for Photodynamic Therapy

Oliver J. Klein[1,*], Hushan Yuan[2*], Nicholas H. Nowell[1], Charalambos Kaittanis[2], Lee Josephson[2], Conor L. Evans[1,ǂ]

1. Wellman Center for Photomedicine, Massachusetts General Hospital, Harvard Medical School, 13th St, CNY149, Charlestown, MA 02129, USA

2. Department of Radiology, Division of Nuclear Medicine and Molecular Imaging, Massachusetts General Hospital, Harvard Medical School, 13th St, CNY149, Charlestown, MA 02129, USA

ǂ Corresponding Author:

Conor L. Evans, Wellman Center for Photomedicine, Harvard Medical School, Massachusetts General Hospital, 149 13th Street, Charlestown, Massachusetts 02129, USA. E-mail: evans.conor@mgh.harvard.edu

*These authors contributed equally to this work.

Synthesis of the EtNBS construct DOTA-Lys(EtNBS)-Lys(PEG_{5K})-β-Ala-Cys(DBCO-PEG_4-cRGD)

Protected L-amino acids, PyBOP and Rink Amide MBHA resin were from Novabiochem (EMD Biosciences). Other special chemicals were from other sources: DOTA(CO_2Bu^t)$_3$ (Macrocyclics), mPEG-NHS ester (5 kDa; Creative PEGworks), Fmoc-Lys(N_3)-OH (AnaSpec), and DBCO-PEG_4-NHS (Click Chemistry Tools). cRGD peptide was cRGDfK from Peptides International. The PDT fluorescent dye ETNBS-acid was synthesized as described.[1] All the other solvents and chemicals were from Sigma-Aldrich. Molecular weights were obtained by MS-ESI Micromass (Waters) and MALDI-TOF analyses at the Tufts University Core Facility. RP-HPLC (Varian ProStar detector and delivery modules) employed an eluent A (0.1% TFA /water) and eluent B (0.1% TFA in 9.9% water in acetonitrile).

1. Synthesis of linker RGD targeting vehicles (**Scheme S1**)

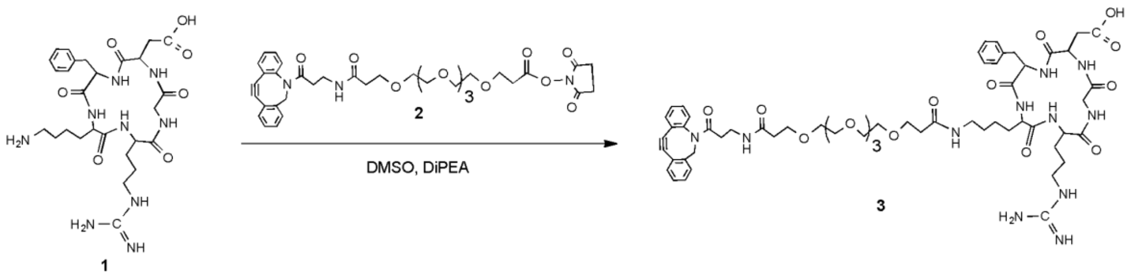

Supplementary Scheme S1. Synthesis of linker RGD targeting vehicles

A stock solution of DBCO-PEG_4-NHS ester (**2**) (containing 7.5mg, 10.8 µmol) in anhydrous DMSO was added to the solution of the RGD peptide, cRGDfK (**1**) (5.6 mg, 9.28 µmol) in anhydrous DMSO (0.4 mL). After DiPEA (9 µL) was added, the mixture was incubated at room temperature overnight. After dilution with buffer A, the mixture was purified by HPLC with gradient of 20%B-100%B in 15 min, then back to 20% B in 5 min and isocratic for 5min; flow: 12 ml/min; wavelength: 226 nm; column: Higgins Analytical Inc. Clipeus C18, 10 µm, 250 x 20 mm, P/N: CS-2520-C181, S/N: 186532. A white powder (**3**) was obtained. Yield: >90% $C_{59}H_{79}N_{11}O_{15}$, MS: expected: 1181.58, found: 11825.

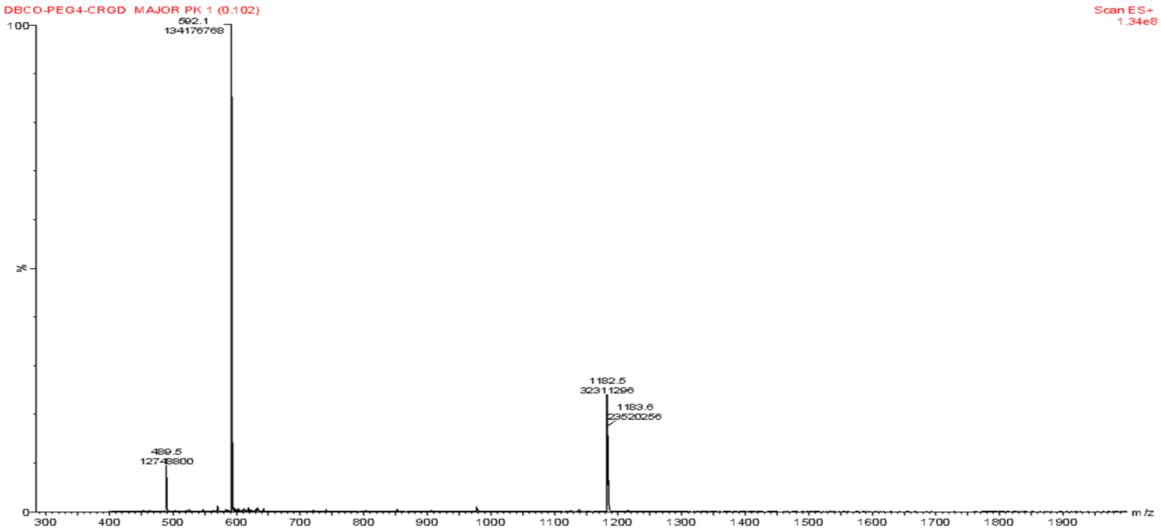

Supplementary Figure 1 Mass spectrum of compound **3**

2. The synthesis of PDT agent: DOTA-Lys(EtNBS)-Lys(PEG-5KDa)-β-Ala-Lys(DBCO-PEG$_4$-cRGD) (**9**) (***Scheme S2***)

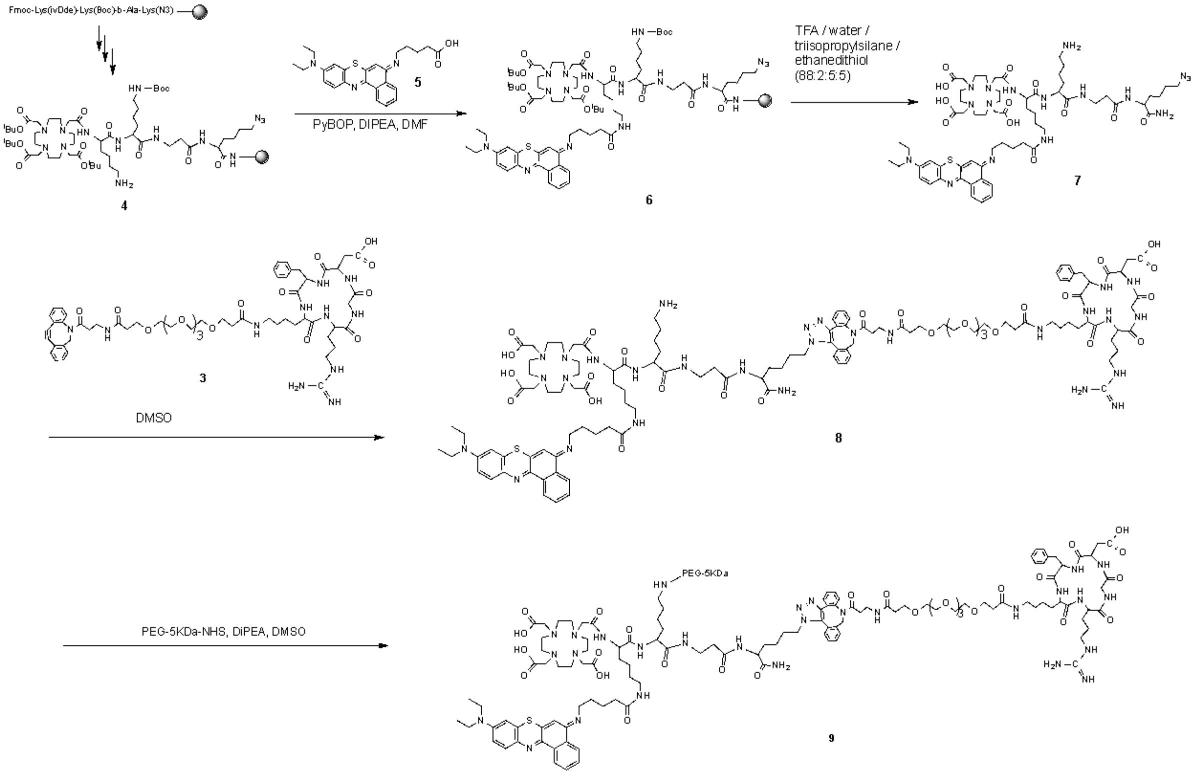

Supplementary Scheme S2. The synthesis of PDT agent: DOTA-Lys(EtNBS)-Lys(PEG-5KDa)-β-Ala-Lys(DBCO-PEG$_4$-cRGD) (**9**).

Synthesis of 7: The DOTA(CO_2-Bu^t)$_3$-Lys(NH_2)-Lys(Boc)- β -Ala-Lys(N_3) peptide (**4**) was

manually synthesized on Rink Amide MBHA resin according to the previous procedure.[2] The attachment of EtNBS was carried out on the solid phase for overnight by using EtNBS-COOH (**5**) (2 equiv.) under the *in situ* activation of PyBOP (2 equiv.) and DiPEA (8 equiv.). Intermediate DOTA-Lys(EtNBS)-Lys(NH_2)- β -Ala-Lys(N_3) (**7**) was released from the solid support with TFA/H_2O/TIS/EDT 88:2:5:5 (twice, 4 h, 20 mL/g resin). The cleavage solution was diluted with 30% acetonitrile in water with 1:1 ratio, then HPLC was employed for further purification with a gradient of 20% B to 100% B in 15 min, back to 20% B in 2min and isocratic for 3min on a C18 column. (Virian: PLRP-S, 100A, 15-20 µm column, flow: 15 mL/min, wavelength : 653 nm). A blue powder of compound (**7**) was obtained after lyophilization with a yield of 14.5%. MS: $C_{62}H_{93}N_{17}O_{12}S$: expected: 1299.69; found: 1300.74 (M+1), 1338.81(M+K^+)

Synthesis of unPEGylated PDT agent (8): *After* Dota-Lys(EtNBS)-Lys(NH_2)-β-Ala-Lys(N_3)NH_2 (**7**) and cRGD-PEG$_4$-DBCO (**3**) (1:1 eq., 0.011 mmol) in DMSO was incubated under room temperature for 1 h., The mixture was diluted by 30% acetonitrile in water with a ratio of 1:1 (v/v) and purified by HPLC on a C18 column (Higgins Analytical Inc. Proto 300 C18 10 µm, 250 x 20 mm, P/N: CS-2520-C181, S/N: 186441) with a gradient of 20%B – 100%B in 15 min, back to 20%B in 2 min and isocratic for 3 min; flow: 12ml/min; wavelength : 653 nm. Yield: ~99%. MS: $C_{121}H_{172}N_{28}O_{27}S$: expected: 2481.27; found: 2482.47 (M+1).

Synthesis of multifunctional PEGylated PDT agent (9): To a solution of DOTA-Lys(EtTNBS)-Lys(NH_2)-β-Ala-Lys(DBCO-PEG$_4$-cRGD)-NH_2 (3.33 mg, 1.34 µmol) in DMSO (0.7 mL), was added the solution of m-PEG$_{5k}$-NHS (20.1mg, 4.02µmol, 3eq). After DIPEA (12.35 µL**,** 71 µmol, 53 equiv. to MSAP**)** was added, the mixture was incubated for 3 days at room temperature. The color of the solution turned from blue to purple. The mixture was neutralized with HOAc before each dilution by acetonitrile and water (0.1% TFA, 1:1 v/v) for HPLC injection. HPLC purification was employed for product purification with a C18 column (Higgins Analytical Inc. Proto 300 C18 5µm, 250X10mm, P/N: CS-2520-C185) under conditions of gradient: 20%-100%B in 20 min, then back to 20% B in 5 min and isocratic for 5 min; flow: 5 mL/min. wavelength: 653 nm. A blue powder was obtained after lyophilization. Yield: 50%, MS: expected: 7483; found: 7530. Only high molecular weight PDT agent (**9**) was determined by FPLC analysis by using an ÄKTA Purifier 10 and SuperdexTM 200 10/300GL column (GE Healthcare) with a running buffer of 0.05 M sodium phosphate, 0.15 M NaCl (0.1% Tween, pH 7.2) and flow rate of 0.8 mL/min.

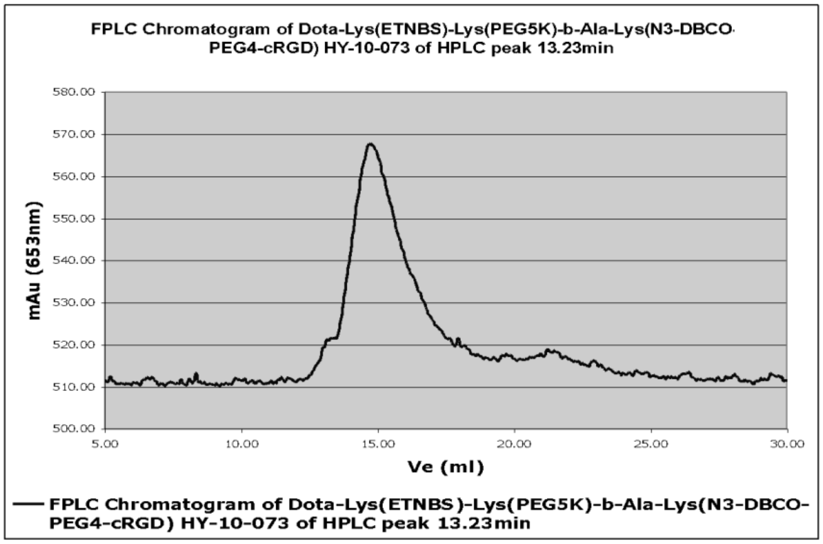

Supplementary Figure 2 FPLC chromatogram of compound **9**

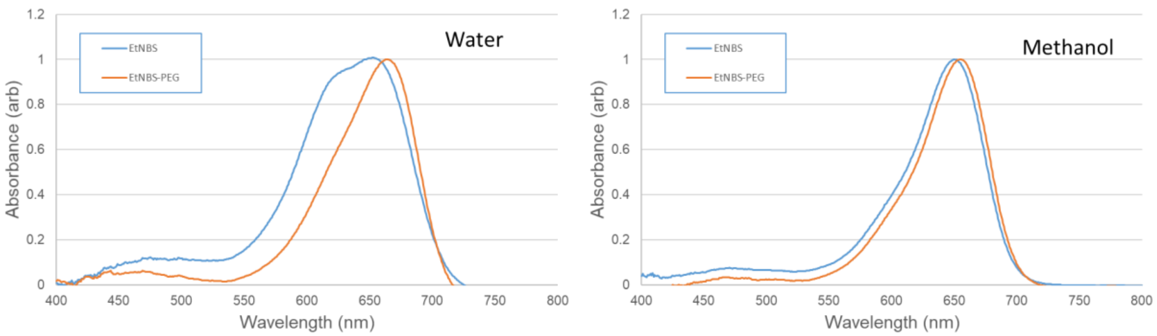

Supplementary Figure 3: Absorption spectrum of EtNBS and the EtNBS-PEG conjugate in water (left) and methanol (right) (5 µM concentration). In methanol, the spectra of both compounds are essentially the same, with the EtNBS-PEG conjugate having a small red solvatochromic shift likely due to the presence of the 5 kDa linear PEG. In water, there is a substantial difference between the molecules: EtNBS displays a blue shift that is typical of a benzothiazinium J-J aggregate. The EtNBS-PEG conjugate shows minimal aggregation, with this protective effect having been afforded by the PEG chain.

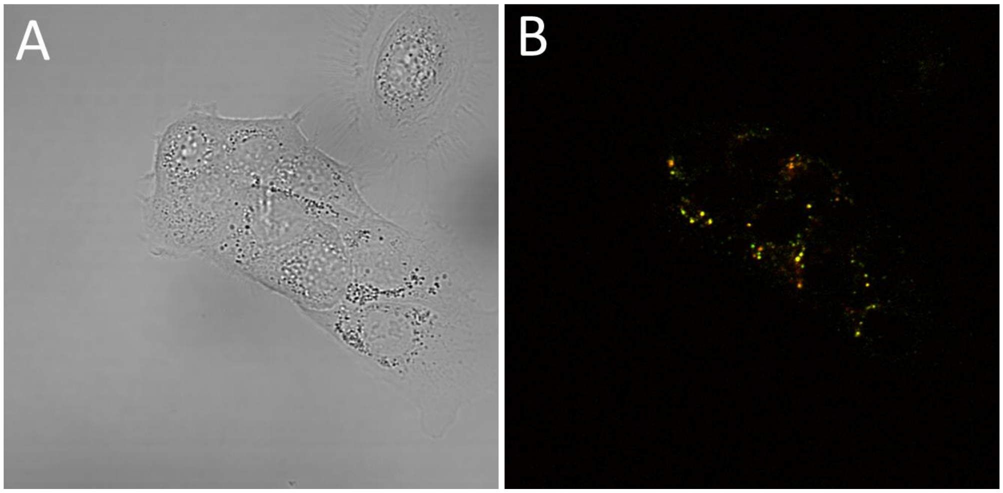

Supplementary Figure 4: Colocalization of the EtNBS-cRGD-DOTA-PEG construct with Lysotracker shows the construct is taken up primarily into the lysosomes. (A) Transmission image showing a single OVCAR5 cell. (B) Confocal microscopy image showing Lysotracker green fluorescence in green and the EtNBS-cRGD-DOTA-PEG construct fluorescence in red. Most lysosomes show a yellow color demonstrating co-localization of the two molecules.

References

1 Verma, S. *et al.* Antimicrobial photodynamic efficacy of side-chain functionalized benzo[a]phenothiazinium dyes. *Photochem. Photobiol.* **85**, 111-118, doi:10.1111/j.1751-1097.2008.00403.x (2009).

2 Guo, Y. *et al.* The PEG-fluorochrome shielding approach for targeted probe design. *J. Am. Chem. Soc.* **134**, 19338-19341, doi:10.1021/ja309085b (2012).
